# ColoNav: patient navigation for colorectal cancer screening in deprived areas – Study protocol

**DOI:** 10.1186/s12885-016-2469-9

**Published:** 2016-07-07

**Authors:** C. Allary, A. Bourmaud, F. Tinquaut, M. Oriol, J. Kalecinski, V. Dutertre, N. Lechopier, M. Pommier, Y. Benoist, S. Rousseau, V. Regnier, V. Buthion, F. Chauvin

**Affiliations:** Hygée Centre, Lucien Neuwirth Cancer Institut- ICLN, CIC 1408 INSERM, 108bis avenue A. Raimond, 42270 Saint-Priest-en-Jarez, France; Gustave Roussy Institut, 114 rue Edouard Vaillant, 94800 Villejuif, France; COACTIS, Lumière University Lyon 2, 16 avenue Berthelot, 69007 Lyon, France; EA 4148 - S2HEP, University Lyon 1/Ecole Normale Supérieure de Lyon, 43, Boulevard du 11 novembre 1918, 69622 Villeurbanne cedex, France

**Keywords:** Colorectal cancer screening, Patient navigation, Realistic evaluation, Implementation research, Health inequalities, Randomized control study

## Abstract

**Background:**

The mass colorectal cancer screening program was implemented in 2008 in France, targeting 16 million French people aged between 50 and 74. The current adhesion is insufficient and the participation rate is even lower among the underserved population, increasing health inequalities within our health care system. Patient Navigation programs have proved their efficiency to promote the access to cancer screening and diagnosis.

**Methods/Design:**

The purpose of the study is to assess the implementation of a patient navigation intervention that has been described in another cultural environment and another health care system. The main objective of the program is to increase the colorectal cancer screening participation rate among the deprived population through the intervention of a navigator to promote the Fecal Occult Blood Test (FOBT) and complementary exams.

We performed a multisite cluster randomized controlled trial, with three groups (one experimental group and two control groups) for 18 months.

**Discussion:**

The study attempts to give a better understanding of the adhesion barriers to colorectal cancer screening among underserved populations. If this project is cost-effective, it could create a dynamic based on peer approaches that could be developed for other cancer screening programs and other chronic diseases.

**Trial registration:**

NCT02369757

## Background

Colorectal cancer (CRC) is the third most common cancer diagnosed and the second leading cause of cancer death in both men and women in France, according to the GLOBOCAN estimates [1]. Approximately, 42 000 new cases were diagnosed in 2012, predominantly men and 17 500 deaths were attributed to CRC [1].

Since 2008, CRC mass screening has been implemented in France. This population-based program targets men and women aged from 50 to 74 who receive a standardized invitation letter, every 2 years, to encourage them to consult their general practitioner (GP). The GP delivers a screening test. People who don’t send their test to the screening center receive a reminder letter. They then receive the test at home within 6 months following the date of the first invitation. The test used is the FOBT, followed by a colonoscopy in case of a positive test result.

Several studies have universally shown that FOBT, followed by colonoscopy in case of a positive test result can reduce CRC mortality [2–6]. But to be efficient, a high participation of the target population is required for these screening programs, at least 50 % [7].

In France, for the period 2009–2010, 17 million French people were invited to participate in the CRC screening program. The national participation rate was only 33.8 % (5.14 million) whereas the national objective was to reach a participation rate of 60 % in 2013. Significant disparities were recorded. The women participated more than the men (36.5 % vs. 31.4 %). Among men, older people (over 70) participated more than the younger ones. Women between 60 and 64 years old, participated more. Moreover, participation rates varied widely according to the regions.

The factors affecting FOBT screening program participation have been analyzed. Gender, age, marital status, educational status, social status and economical status can influence participation [8]. In France, the correlation between socioeconomic status and participation rate has been observed [9].

It is interesting to note that France is one of the Western European countries where socioeconomic gradient – measured by the educational status – has the highest impact on mortality [10]. Moreover, social inequalities impact cancer rates, including colorectal cancer [11]. As a matter of fact, France is one of the European countries where educational status, among all risk factors considered, has the highest impact on cancer mortality.

Patient Navigation (PN) is a patient-centered healthcare service delivery model that centers on reducing barriers to cancer care [12–15]. The first “Patient Navigation” program was established in New York in the early 1990s, by Freeman, a surgical oncologist at Harlem Hospital [15]. The development of the PN concept was related to the findings of the American Cancer Society National Hearings on Cancer in the Poor (Program No Need to Die). Based on these hearings, the first PN program was built. It focused on the critical window of opportunity to save lives from cancer by eliminating barriers to timely diagnosis and treatment for low-income populations [16]. The program was composed of navigators who were from the community or culturally similar to the population served. The patient navigator was defined as an individual who could educate and empower patients, serving as their advocate in navigating the health care system. This intervention succeeded in improving access to breast and colorectal cancer screening for the deprived population. It helped improve survival rates by 5 years [17, 18]. Moreover, this approach is considered as an effective means to reduce inequalities in the USA [19]. These interventions have been increasingly adopted throughout the Unites States, Canada [20] and China [21, 22].

We hypothesize that inequalities generated by general prevention measures can be offset with specific measures, thanks to peer education which targets population from underserved areas.

The primary objective of this study is to determine effectiveness of a PN Program on CRC screening participation among the underserved population.

The secondary aims are: 1) to perform a realistic evaluation of an innovating intervention, of which the effectiveness has been demonstrated by several randomized studies in another health care system 2) to assess the implementation into a specific context 3) to identify factors favoring or hindering the effectiveness of the intervention.

The purpose of this article is to describe the protocol of this study.

## Methods/Design

### Study design

COLONAV is a population-based cluster randomized control study. It assesses the effectiveness of an intervention previously described and evaluated in another cultural environment and in another health care system, as regards CRC screening attendance.

Three parallel groups will be compared:

- one experimental group with intervention
- two control groups without intervention

### Selection of study sites and participants

The study will include men and women aged from 50 to 74 years old, invited to screening and living in underserved areas. The research will be conducted in the following five districts because of their different socioeconomic characteristics:

- A rural district (L’Ardèche, 313 578 inhabitants)
- A district with an industrial economy and low development (La Loire, 746 115 inhabitants)
- A district with a tertiary economy and high development (Le Rhône, 1 708 671 inhabitants)
- A district where CRC screening has been implemented for a long time (La Côte d’Or, 524 144 inhabitants)
- One of the suburban districts of the capital (Le Val-de-Marne, 1 318 537 inhabitants)

In each district, the geographical areas to be studied will be determined by matching the criteria in terms of the socioeconomic indicators and the low participation rate for CRC screening. In France, each district is divided into IRIS zones, ie aggregated units for statistical information used as a system for dividing the country into units of equal size (2000 residents per basic unit). We will use the French European Deprivation Index (EDI) [23] to determine the most deprived IRIS zones (quintiles 4 and 5 of the Index corresponding to the most disadvantaged areas) and the participation rates of each zone to select the lowest ones.

### Sample size

We estimated that the intervention would be relevant if it provided an 8 % absolute increase in the colorectal cancer screening participation rate (22 % in the control group and 30 % in the intervention group). With a two-sided α risk of 0.05 and a β risk of 0.1, and assuming an intraclass correlation coefficient for clusters of 0.004 and a mean of 500 patients per cluster (assuming an average of 2000 people per IRIS zone and percentage of people between 50 and 75 years equal to 25 % of the total population), we needed 4 clusters per group (2000 people per group). Taking into account the three groups (one intervention group and two control groups) and stratified by department gives a total number included about 30 000 individuals.

We establish that the number of IRIS zones needed to meet the objectives of the study is 20 intervention IRIS zones and 40 control IRIS zones [24].

So, for each district, we will randomly select:

- four contiguous intervention IRIS zones
- eight control IRIS zones divided into two groups (one adjacent and one distant from the intervention group)

### Study groups

#### Patient navigation intervention

**Fig. 1:**
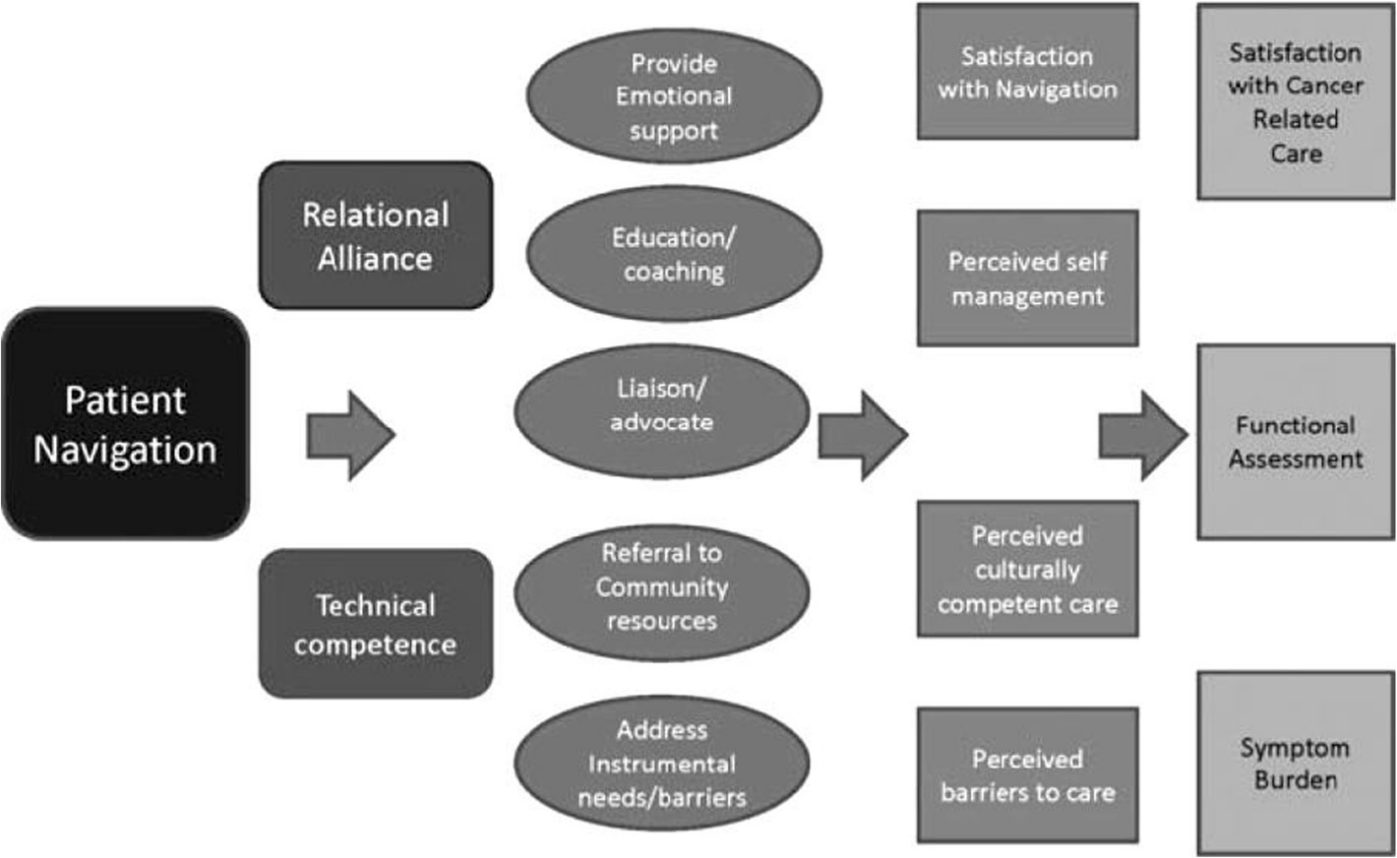
Conceptual model of patient navigation (Fiscella 2011)

The conceptual model of PN intervention was developed by Fiscella [25] to articulate the relations between program inputs, navigation activities, and specific outcomes. In this model, the author distinguished two domains of navigator’s services: instrumental/logistical, reflecting technical competence, and interpersonal/educational, reflecting the relational alliance [26] (Fig. 1).

The main role of the navigators is to promote CRC screening among the target population to increase participation rates in these areas. The missions of the navigators are to contact individuals living in the intervention area and invited by the screening structure and to guide them to the realization of the screening test.

Navigators’ activities will be: 1) to establish partnerships with local associations, religious places, health centers, health fairs, GPs… in order to participate in local events and to meet the target population 2) to find a committee room to meet people who are in need 3) to communicate by phone or face-to-face with the target population 4) to provide personalized assistance to each person in order to help them to overcome obstacles in the realization of the screening test (psychological, financial or logistical barriers) 5) to collect patient socio-demographic information.

#### Control group

Men and women in the control group will receive the invitation and the usual standard information document. The invitation is an administrative letter sent by all the screening management structures when men and women are scheduled to be invited to participate in the national screening program; at 50 years old and then every two years.

### Study protocol

#### Local diagnosis

We will diagnose local needs in the five selected districts before the intervention. A research team conducted semi-structured interviews with relevant stakeholders (individuals representing the target population, GPs, local actors of associations) in order to accurately assess the needs of the target population. The goal of this diagnosis was to have a better understanding of barriers and obstacles of the target population but also of the different stakeholders about the screening.

#### Recruitment and training of navigators

The strategy is to recruit and train one navigator per district. Candidates’ recruitment will be done by means of job description that will be widely diffused. The profile desired is a peer, i.e. a person corresponding to the target population of the study, living in the intervention zones. The district committees of “La Ligue contre le Cancer” (a French non-governmental organization which promotes cancer screening, gives support to patients and funds research, will be in charge of recruiting each navigator). Navigators will receive formal training for one week on all issues related to the following: the navigation process, cancer generalities, CRC screening, counseling and support to patients as needed.

#### Patient navigation intervention

Navigators will be hosted by the district committees of “La Ligue contre le Cancer” during the intervention (18 months). In each of these structures, a supervisor will be assigned to each navigator. The intervention will mainly consist of: outreaching the target population and accompanying the target population to be screened.

In order to gather information about the navigators ‘experiences (their good practice and challenges they are facing) meetings will be organized with the researchers of the study. Navigators will regularly update a field journal and a database. They will participate in the practice exchange days and develop quarterly progress reports.

#### Realistic evaluation

In this study we will use a realist evaluation approach, based on Pawson and Tilley’s work [27]. This theory-driven approach focuses evaluation on the study of what works, for whom and in what circumstances. These relationships are constructed as Context-Mechanism-Outcome configurations [28]. Considering the evaluation design, multidisciplinary teams will be associated (public health teams, human and social sciences teams and economic research teams.).

Both quantitative and qualitative data will be collected to perform the realist evaluation.

### Outcomes assessments

#### Primary outcome

To assess the effectiveness of the intervention, the primary outcome will be measured by the difference between before/after participation rates in the intervention zones and in the control zones. Other criteria will be used according to the recommendations made by the National Patient Navigation Leadership Summit (NPNLS) and published in Cancer in 2011 [29]: time to participate, participation in the whole procedure, time to complete the colonoscopy after a positive FOBT test result.

Eighteen months after the beginning of the intervention, each screening management structure, in charge of sending the invitation to individuals included in the mass screening program, will collect the participation status of the men and women included in the study. Each district structure has a database with the contact details of all individuals, eligible to receive an invitation to the national screening program. After the beginning of the intervention, the 18 month-participation rates will be measured, in selected areas. The following items will be measured: program coverage rate (percentage of people guided by navigators), screening participation rate, percentage of patients who get screened after the navigator intervention, colonoscopy completion rate.

#### Secondary outcome

The secondary outcomes for this study include 1) context of patient-related evaluation, as recommended by the Patient-Reported Outcomes Working Group (PROWG) of the Navigation program [30] 2) evaluation of evidence-informed practice 3) ethical evaluation 4) cost-effectiveness 5) organizational analysis.

A patient-related context evaluation will use measures reported by the target population in contact with navigators according to the PROWG [25]. The following fields will be explored: habits and fears regarding cancer and screening, cultural aspects, satisfaction about the intervention. An approach with questionnaires will be used, completed by semi-structured interviews. Given the lack of validated questionnaires in French, we will use a non-validated questionnaire.

In view of a future implementation of the program after its evaluation and to identify variances with the initial program, a researcher will perform an ethnographic evaluation. In total, 10 days of tracking will be done in order to know the detailed practice of the navigators, including the way they behave in order to adapt to local context for two reasons: (a) to take into consideration the potential future implementation of the program after its evaluation; b) to identify the potential variances with the initial program.

An ethical evaluation will include focus group and interviews with navigators lead by a team specializing in ethics of public health. Indeed, a project to correct social inequalities in health, through the intervention of peers, raises at least two ethical issues: 1) justice-related challenges: if justice is the guiding value of the intervention that aims to tackle health disparities, it can also trigger potentially unfair identity assignment and stigmatization. 2) Such a community-based intervention also raises challenges regarding the trust-building process.

An economic assessment of the intervention will investigate the cost-effectiveness of this program.

An organizational analysis will take the recommendations of the US program of navigation into account, to ensure comparability with other interventions [31]. The objective is to determine the conditions of implementation, sustainability and reproducibility of the program. The method of investigation is a qualitative method. Observational data will be collected at different moments: preparation of the intervention, recruitment of the navigators, during and after the intervention.

### Statistical analysis

#### Quantitative analysis

All collected individual variables will be described by frequency (%) for categorical variables, and mean (SD) and median (Q1 -Q3) for quantitative variables. The data will be summarized separately in two tables: one for the period before intervention, and another for the period after intervention. The effectiveness of the intervention will be measured comparing the delta of before-after participation rate in intervention area and control areas. Completion time of the exams (screening test and colonoscopy) between intervention areas and control areas before and after the intervention will also be compared. The difference in participation will be analyzed by logistic regression analysis methods taking into account the interactions between the period and area. The influence of other collected variables will be tested using univariate tests (Chi2 or Fisher test for categorical variables and Student or Wilcoxon test for quantitative variables). A multivariate model will be implemented in order to take possible confounding factors into account. Analysis of participation time will be done in two ways: firstly by taking the censored data with a Cox model into account and secondly with a ANOVA model (on the subgroup of patients who participate). The level of statistical significance will be of 5 %.

#### Qualitative analysis

First, recorded interviews will be transcribed in verbatim. Transcripts will be read holistically, and then line-by-line to extract significant statements from the interviews, following established guidelines for a thematic analysis. These statements will be used to generate specific codes, and each transcript will then be coded using this thematic coding scheme. The themes emerging from the first interviews will help to refine the interview guide used for the next set of interviews. The latter will in turn be used to inform the next set and so on. Data analysis will be performed simultaneously and continually with the data collection, in order to identify the arising of data saturation. The gathered information will then be categorized into five main themes, based on the objectives of the study.

#### Cost effectiveness analysis

In order to estimate the cost-effectiveness of the implementation of such a program, marginal costs, with encryption on the actual cost, observed during the installation, as well as a projection on a routine cost will be taken into account.

#### Organizational analysis

The content of the interviews with navigators and institutional partners will be subject for a qualitative analysis by the IRaMuTeQ software. A statistical analysis of different texts will be performed in order to generally describe the corpus (number of texts, occurrences, of forms …). This will determine the navigators’profiles, as well as a hierarchical organizational structure. It will also create a theoretical model for action.

## Discussion

This study aims: 1) to implement a PN intervention for which the efficacy has been demonstrated in a different context 2) to assess the effectiveness of the intervention in this context 3) to identify the context characteristics that may interact with the effect of the intervention (factors favoring or hindering). The ColoNav project should make it possible to determine if the patient navigation program is suitable and if it works in the context of the French healthcare system. It should allow us to identify the effects of the context and to give details concerning the specific favoring and hindering factors. Finally, ColoNav will allow the creation of a new enhanced program. If this intervention is estimated as being cost effective, it could create new dynamics for peer approaches. The latter could be developed for the screening program of other cancers or other chronic diseases.

## Abbreviations

CRC, colorectal cancer; EDI, European Deprivation Index; FOBT, fecal occult blood test; GP, general practitioner; INCA, Institut National du Cancer; NPNLS, National Patient Navigation Leadership Summit; PN, patient navigation; PROWG, Patient-Reported Outcomes Working Group
